# Gene Expression Correlates with the Number of Herpes Viral Genomes Initiating Infection in Single Cells

**DOI:** 10.1371/journal.ppat.1006082

**Published:** 2016-12-06

**Authors:** Efrat M. Cohen, Oren Kobiler

**Affiliations:** Department of Clinical Microbiology and Immunology,Sackler School of Medicine, Tel Aviv University, Tel Aviv, Israel; Emory Vaccine Center, UNITED STATES

## Abstract

Viral gene expression varies significantly among genetically identical cells. The sources of these variations are not well understood and have been suggested to involve both deterministic host differences and stochastic viral host interactions. For herpesviruses, only a limited number of incoming viral genomes initiate expression and replication in each infected cell. To elucidate the effect of this limited number of productively infecting genomes on viral gene expression in single cells, we constructed a set of fluorescence-expressing genetically tagged herpes recombinants. The number of different barcodes originating from a single cell is a good representative of the number of incoming viral genomes replicating (NOIVGR) in that cell. We identified a positive correlation between the NOIVGR and viral gene expression, as measured by the fluorescent protein expressed from the viral genome. This correlation was identified in three distinct cell-types, although the average NOIVGR per cell differed among these cell-types. Among clonal single cells, high housekeeping gene expression levels are not supportive of high viral gene expression, suggesting specific host determinants effecting viral infection. We developed a model to predict NOIVGR from cellular parameters, which supports the notion that viral gene expression is tightly linked to the NOIVGR in single-cells. Our results support the hypothesis that the stochastic nature of viral infection and host cell determinants contribute together to the variability observed among infected cells.

## Author Summary

Single cell variation is of major interest in understanding key biological processes, like cancer, development and host pathogen interaction. During viral infection, these cell to cell variations can change the outcome of the whole organism infection. We suggested that differences in the number of parental viral genomes that initiate the replication process alter the outcome of infection among single cells. In this work we present a method based on genetically barcoded herpesvirus recombinants to identify the number of viral genomes initiating replication in individual cells. Our results indicate that viral gene expression is tightly linked to the number of viral genomes replicating per cell. Remarkably, we found that high cellular gene expression was an indicator for a lower viral gene expression in a given cell. We suggest that variations among single cells result from preexisting differences among cells, as well as from random viral host interactions.

## Introduction

Cell-to-cell variability is an important factor in cancer, development, evolution, host-pathogen interactions and other key biological processes [1–3]. The variability observed among single cells mainly arises from deterministic factors, i.e. preexisting molecular regulatory mechanisms [3, 4]. In the context of viral infections, it was suggested that stochastic interactions between a virus and individual host cells could contribute to variability in the outcome of infection in the entire infected organism [5–9]. Much of the variation in the outcome of infection can be attributed to the specific cell state prior to infection [10]. Here, we provide evidence that the viral gene expression level also depends on the actual number of viral genomes initiating the infection process.

Genetically barcoded viruses are used for studying cellular clonality (see for example: [11–13]); however, only a few studies have used genetic barcoding of viral genomes to study viral properties. Barcoded RNA viral genomes were used to identify bottlenecks in viral diversity, both inside the infected host [14, 15] and during transmission among hosts [16]. Thus, barcoding of viral genomes can be a useful tool in studying bottlenecks during viral replication, even on the single cell level [17].

Herpes simplex virus 1 (HSV-1), a large DNA virus, is a very common human pathogen that causes significant morbidity throughout the world. HSV-1 is part of the large family of herpesviridae, and its replication has been studied as a model for the entire family of viruses. To replicate, naked herpes genomes enter the nucleus. Upon entry, the naked viral DNA associates with host histones to form nucleosomes. These chromatin structures are regulated by host histone modifying enzymes and are essential both for the lytic and the latent viral infection pathways [18, 19]. Recent studies suggest that these interactions are cell type specific [20, 21]. Interactions between the viral DNA, the tegument protein VP16 and host factors determine the probability of initiating immediate early gene expression [22]. Immediate early proteins activate expression of early and late genes and counteract host defense mechanisms. Both intrinsic and innate immunity are inhibited by the viral immediate early protein, ICP0 [23]. The requirement for ICP0 function varies between different cell types [24, 25]. Thus, HSV-1 closely interact with the host cells, and specific mechanisms in the host cells can modify the outcome of the infection.

Following early gene expression, viral replication initiates in specific domains known as pre-replication compartments [26]. These small structures grow in size, move and coalesce to form replication compartments (RCs) [26, 27]. On the other hand, only one parental genome can be found in each RC [28, 29]. The number of RCs was reported to be limited [26, 30]. A bottleneck, limiting the number of incoming herpes genomes that are expressed and replicated, was observed; this number was estimated to be less than 10 per cell (even in multiplicity of infection 100) [29, 31, 32].

To test if viral gene expression is dependent on the number of incoming viral genomes replicating (NOIVGR) per cell, we developed an HSV-1 genetic barcode system that estimates the NOIVGR at the level of single cells. Our system is based on DNA barcoding of mCherry expressing HSV-1 recombinants and identification of the progeny barcoded recombinants derived from a single cell. We determined that the mCherry fluorescence levels are surrogates for viral gene expression and found fluorescence levels to correlate with the NOIVGR. We developed a model that predicts the NOIVGR according to cellular parameters. We conclude that cellular determinants and stochastic effects of NOIVGR determine the outcome of infection in individual cells.

## Results

### Construction of 14 barcoded viral recombinants

To study single cell variables that determine herpesvirus gene expression, we developed a system based on isogenic genetically-tagged recombinants of HSV-1 strain 17. Fourteen unique sequences were inserted into a pOK11 plasmid [32] downstream to the mCherry gene (see Methods). Using homologous recombination, these short sequences and the mCherry gene were inserted into HSV-1 genomes, resulting in 14 DNA-barcode tagged viruses. All 14 recombinants showed comparable growth curves (S1 Fig) and were verified by qPCR. The genome from each virus can be specifically identified by the barcode sequence (S2 Fig).

### Fluorescence expression indicates viral gene expression

**Fig 1:**
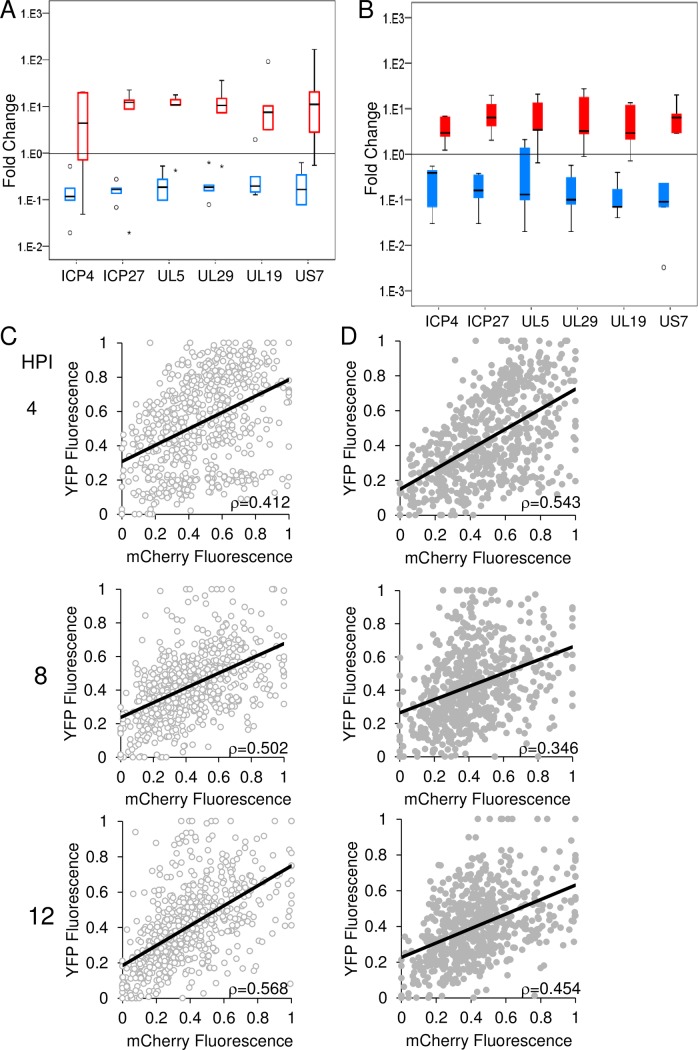
Relative fluorescence intensity of infected cells correlates with viral gene expression. (A-B) Cells were infected with the barcode-viruses at MOI 10 (A) or 100 (B). At 3 HPI, the cells were sorted into three groups according to their fluorescence intensity from lowest (blue 30% of total), through middle (base line levels 40%) and highest (red 30%). Total RNA was collected from each group and RT-qPCR was performed for several viral genes as indicated. In each experiment, expression levels from highest and lowest groups were normalized to that in the middle group. Box plots of five biological repeats generated by SPSS are presented. Outliers (1.5<box length<3) are marked with circles, and extreme scores (3<box length) are marked by star shapes. (C, D) Vero cells infected with OK21 viral recombinants at MOI 10 (C) and MOI 100 (D) were visualized during the infection at the indicated time points. The graphs represent the relative yellow fluorescence (mVenus) vs. the relative red fluorescence (mCherry). Each graph represents 3 biological replicates, in each replicate three frames were analyzed. A total of 671 and 639 cells were plotted for MOI 10 and MOI 100, respectively. A trend line that was calculated using the ordinary least squares (OLS) method and the measured Pearson correlation, are presented in each graph.

We hypothesized that the measured mCherry fluorescence expressed from the CMV promoter cloned into the viral genome is a surrogate for the expression of native viral genes. To test this hypothesis, we sorted Vero cells infected with barcoded viruses at MOI 10 or 100 into three populations according to their fluorescence (30% lowest, 40% middle and 30% highest of the total population). Viral gene expression levels of immediate-early (ICP4, UL54-ICP27), early (UL5, UL29) and late (UL19, US7) genes were measured by RT-qPCR from each population. We compared expression of the lowest and highest populations to the middle subpopulation (Fig 1A and 1B). As expected, in the highest fluorescence population all viral genes were expressed at a higher level than in the middle subpopulation. Similarly, in the lowest fluorescence population, viral genes were expressed at lower levels.

To ensure that the correlation between the mCherry expression from the CMV promoter to the viral gene expression is maintained throughout the infection process, Vero cells were infected with a dual color expressing virus (OK21) which carry the mCherry expressed from the CMV promoter and the mVenus (a yellow fluorescent protein) fused into the late gene UL25 under its native promoter. We monitored the infection, and at representative time points, we estimated the mCherry and YFP fluorescence levels (Fig 1C and 1D). Our results indicate a significant continuous correlation between the levels of the mCherry and YFP expressed from the late viral promoter. Together with RT-qPCR results, we conclude that the fluorescence expressed from the CMV promoter in viral genomes is a surrogate for viral gene expression during the entire infection process.

### Relative fluorescence is maintained throughout infection

**Fig 2:**
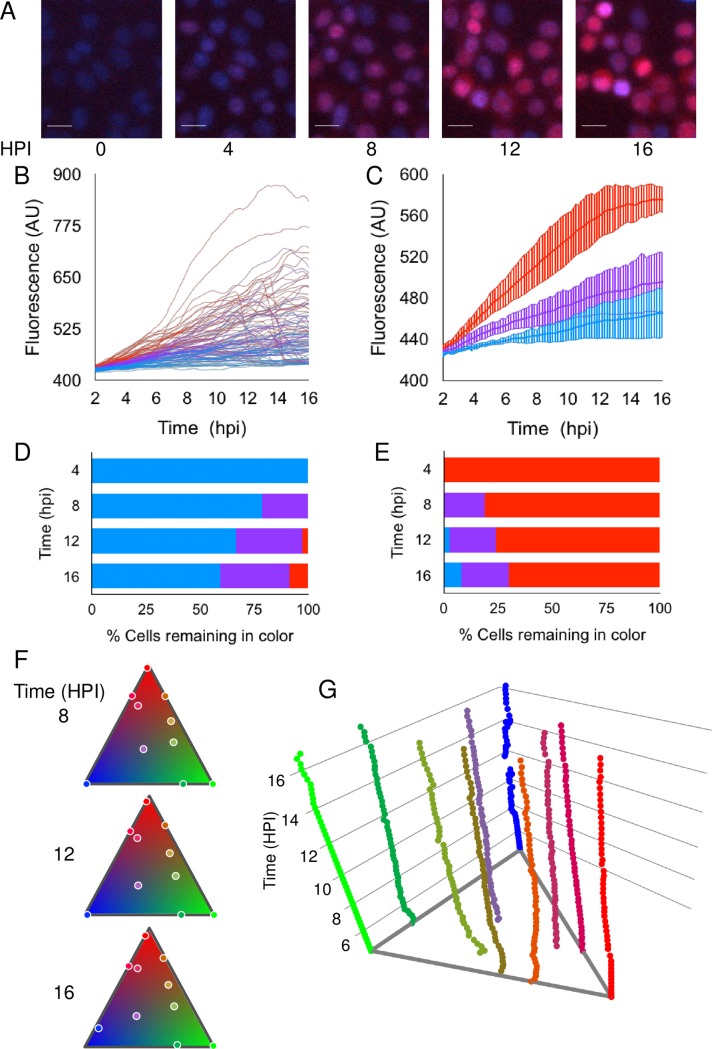
Relative fluorescence is maintained throughout infection. (A-E) Cells infected with a mixture of the barcode-viruses at MOI 100 were monitored for 18 hours. (A) Snapshots of cells at different time points (as indicated) are presented. Scale bar 20μm. (B-E) The infected cells were divided according to the fluorescence intensity of the cells at 4 HPI, from lowest (blue 30% of total), through middle (purple 40%) and highest (red 30%). (B) The fluorescent profile of 97 individual cells from a representative single well (4 different frames) is presented as a function of time. (C) The mean of the cell fluorescent profiles, for each of the groups of fluorescence intensity, was calculated for each well. A mean of three wells (with the standard deviation between the wells-stripe lines) is presented. (D-E) At each time point indicated, cell profiles were sorted according to fluorescence levels. The 30% lowest population (D) and 30% highest population (E) were compared to the 4 HPI time point. Each bar represents the ratio of cells from the 4 HPI segregation (colored as above), as found at the indicated time point. An average of the ratios from three different wells is presented. (F-G) Cells were infected with three different fluorescence expressing viral recombinants (each expressing one of the following fluorescent genes: mCherry, EYFP or mTurq2) at MOI of 100. Ten representative cells were plotted according to their hues on triangular barymetric plot. Each vertex represents a different pure color (red, blue and green), edges represent a combination of two colors and the inside of the triangle represent mixture of three colors. (F) The ten cells (dots) are shown at 3 HPI as indicated. (G) A three dimensional plot of the changes in hue over time (z-axis) for the ten cells (lines) is presented. Each cell is plotted in a representative color.

To test if the relative mCherry fluorescence level measured at a given point in time represents the relative fluorescence level throughout the infection, we followed infected Vero cells for 16 hours under a fluorescence microscope. The movies obtained (Fig 2A, S3A Fig and S1 and S2 movies) suggest that cells expressing fluorescence early, maintain a high level of fluorescence throughout the infection. To quantify this phenomenon, we plotted the fluorescence level from each cell at each time point (Fig 2B and 2C and S3B and S3C Fig). We categorized the cells according to the fluorescence level at 4 hours post infection (HPI) (30% of the cells with the highest or lowest fluorescence). In most of the cells, the level of fluorescence did not change throughout the infectious cycle (Fig 2D and 2E and S3D and S3E Fig). At MOI 100, less than 10% of the cells that were in the lowest fluorescence group at 4 HPI, switched to the highest fluorescence group at 16 HPI, and vice versa. At an MOI 10, this distinction was less prominent, due to the low fluorescence level at 4 HPI. We noticed that at 16 HPI some of the cells showed cytopathic effects, and moved from the focal plane, a phenomenon that may account for some of the movements between the groups. These results indicate that the relative fluorescence level throughout the infection is a consistent parameter of an infected individual cell. The implication is that relative fluorescence levels can be represented by a single measurement during the infection, as in our experimental system described below.

To ensure that we identify all viral genomes expressing and replicating in a given cell, we monitored color variation of cells during infection with three different fluorescence expressing recombinants. The recombinant viruses (OK11, OK12 and OK22) express mCherry, EYFP and mTurq2, respectively under the CMV IE promoter [32], [33]. Vero cells were infected with an even mixture of the three recombinants at MOI 100 and were visualized for 16 hours under a fluorescence microscope. Approximately 5 HPI (the time it takes for all fluorescent proteins to express, fold and accumulate to detectable levels), each cell obtained a specific hue that remained relatively stable the entire infection time (Fig 2F and 2G, S3 Movie). Notably, while the cell became brighter in time, the proportion of the different colors remained similar. These results suggest that expression is initiated and maintained throughout the infection from the same input viral genomes. As we previously showed that expressing genomes are also the ones replicating [31], we conclude that most incoming viral genomes, that initiate replication, are detectable.

### Experimental barcode system identifies individual replicating HSV-1 genomes in single cells

**Fig 3:**
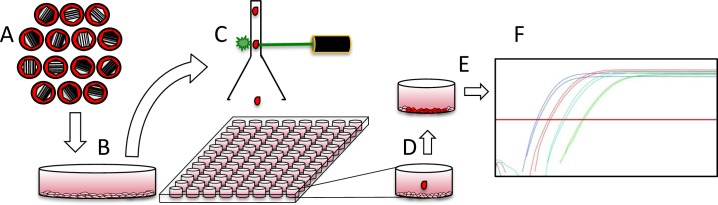
Experimental system to identify replicating HSV-1 barcodes in single cells. A schematic illustration of the steps undertaken in a single cell experiment. (A) Genetically-tagged HSV-1 viruses were evenly mixed and used to inoculate Vero cells (B) The infected cells were incubated for 3 hours, then detached from the plate and transferred to a sorting flow cytometer. (C) The cells were sorted according to their fluorescence intensity into a 96-well plate. (D) Each well of the 96-well plate was pre-seeded with uninfected Vero cells, such that the sorted infected cell was dropped onto a layer of Vero cells. (E) At 24 and 48 HPI, each well was scanned with a fluorescence microscope to discover a single-focus plaque per well, which was further characterized. (F) At 144 HPI, the cells were lysed and analyzed with qPCR to discover the identity and number of barcode progeny.

We hypothesized that cell-to-cell variation might result from differences in the number of incoming viral genomes replicating (NOIVGR) in each cell. To test this possibility, we developed a method to assess the NOIVRG in individual cells. Cells were infected with a mixture of fourteen barcoded viruses (Fig 3A). The mixture contained equal quantities of plaque forming units (PFU) from each virus. The cells were infected at MOI either 10 or 100 (Fig 3B). At 3 HPI, the cells were collected and sorted according to their fluorescence intensity (Fig 3C). Individual cells were sorted onto a pre-seeded 96-well plate (Fig 3D) and were allowed to develop into infectious centers. At 144 HPI, the infectious center spread to most of the cells in the well (Fig 3E). The contents of each well were collected, lysed and analyzed with qPCR, to determine the identity of the progeny barcodes (Fig 3F).

**Fig 4:**
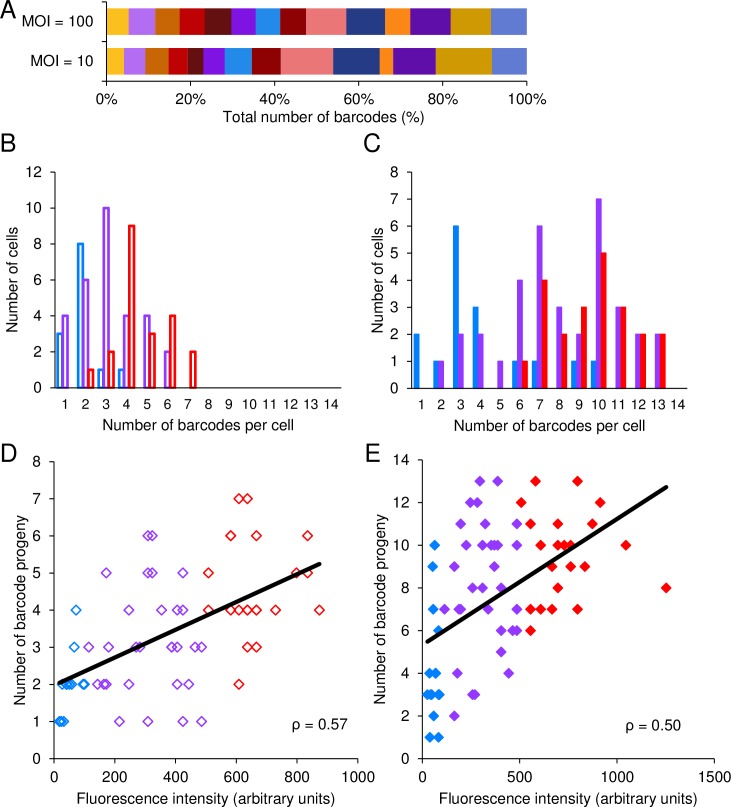
The number of barcodes replicating in individual Vero cells correlates with fluorescence. (A) The relative proportion of each barcode of the total number of barcodes detected from all infectious centers analyzed. The results are presented according to MOI, and each barcode is represented by a different color. (B-E) The output infectious centers, each originated from one individual infected cell, were lysed and analyzed with qPCR. A total of 137 cells from three experiments are presented. (B, C) The distribution of the number of barcodes per cell is depicted at MOI 10 (B, open bars) and MOI 100 (C, solid bars). Each MOI is categorized according to the fluorescence intensity of the cells, from low (blue), through intermediate (purple) and high (red). (D, E) The number of replicated barcode progeny from individual cells, infected at MOI 10 (D) and MOI 100 (E), was plotted against expressed fluorescence, as measured by the cell sorter. Each point is color coded as above. A trend line that was calculated using the ordinary least squares (OLS) method and the measured Pearson correlation, are presented in each graph.

First, we infected Vero cells and isolated single cells as described above. The number of barcodes detected in each well at MOI of 100 ranged between 1 and 13; and at MOI of 10, between 1 and 7 (Fig 4B and 4C). These results suggest that the barcodes detect the NOIVGR in the initial infected cell; and corroborate our previous estimate of the number of expressed herpes genomes inside a cell [31, 32].

We hypothesized that progeny barcode viruses derived from the infectious center represent the replicated parental viruses in the original single Vero cell. We ensured that barcoded recombinants have similar growth properties (S1 Fig). Fig 4A shows that at each MOI all fourteen barcodes can be detected by qPCR (a collective output from all the wells tested). Moreover, the distribution of all the barcodes after an MOI 100 was similar. At MOI 10, the distribution was noisier, probably reflecting the low number of barcodes per cell at this MOI, and the subsequent stochastic effects. Taken together, these results suggest that the recombinant viruses outcompete each other randomly per cell.

### The number of barcoded genomes replicating in individual cells correlates with fluorescence

Sorting the cells enables measuring the forward scatter (FSC is usually considered as a measure of cell size), side scatter (SSC is usually considered as a measure of cell granularity) and fluorescence of each individual cell. We evaluated the relationship of these parameters to the NOIVGR (S5 Fig). Our initial results indicated that MOI and fluorescence were dominant predicting factors for the number of parental genomes that replicated in Vero cells. To characterize the role of MOI in predicting the NOIVGR, we performed all experiments with MOI of 10 or 100. We found that the average number of replicating viral genomes, as indicated by the mean number of barcodes detected by qPCR, is dependent on MOI (for MOI of 10, the mean was 3.4, and for MOI of 100, 7.6, p< 0.001) (Fig 4B and 4C).

To evaluate the correlation between fluorescence and the NOIVGR, we sorted cells according to their fluorescence: low, intermediate and high (S4A Fig). Cells expressing lower fluorescence replicated a smaller number of viruses than did those expressing intermediate or higher fluorescence (Fig 4B and 4C and S1 Table 1). At MOI 10 (Fig 4B), differences between the high fluorescent cells and cells of either low or intermediate fluorescence were statistically significant (p < 0.001). Moreover, the difference between the low and intermediate groups was also statistically significant (p < 0.04). At MOI 100 (Fig 4C), differences between low fluorescence and the other two categories were statistically significant (p < 0.001); however, the difference between the intermediate and high groups was minimal. We accumulated 64 cells infected at MOI 10, and 73 cells at MOI 100; and plotted the number of barcoded progeny against the level of fluorescence (Fig 4D and 4E). We calculated the Pearson correlation between these parameters for each MOI (0.57 and 0.50 for MOI 10 and 100, respectively, p < 0.001 for both MOIs). Taken together, these results indicate that the MOI and the level of fluorescence emitted by infected cells enable estimating the NOIVGR in single cells with a high degree of confidence.

### The correlation between the number of barcodes replicating in cells and viral fluorescence is maintained in individual human fibroblasts

**Fig 5:**
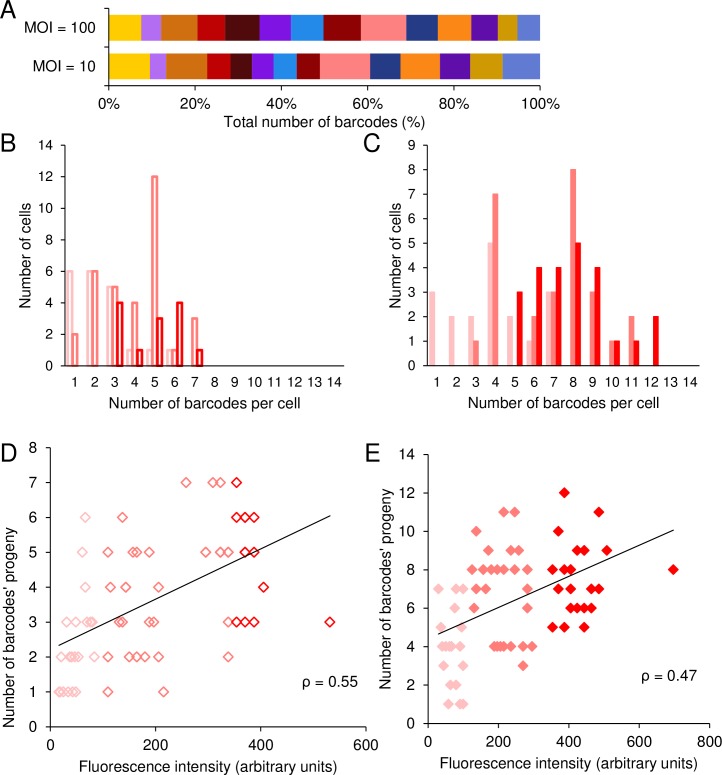
The correlation between the number of barcodes replicating in cells and viral fluorescence is maintained in individual human fibroblasts. (A) The relative proportion of each barcode of the total number of barcodes detected from all infectious centers analyzed. The results are presented according to MOI, and each barcode is represented by a different color. (B-E) The output infectious centers, each originated from one individual infected cell, were lysed and analyzed with qPCR. A total of 135 cells from three experiments are presented. (B, C) A distribution of the number of barcodes per cell is depicted at MOI 10 (B, open bars) and MOI 100 (C, solid bars). Each MOI is categorized according to the fluorescence intensity of the cells, from low (pink), through intermediate (light red) and high (red). (D, E) The number of replicated barcode progeny from individual cells, infected at MOI 10 (D) and MOI 100 (E), was plotted against expressed fluorescence, as measured by the cell sorter. Each point is color coded as above. A trend line that was calculated using the ordinary least squares (OLS) method and the measured Pearson correlation, are presented in each graph.

To test whether the correlation between fluorescence and the NOIVGR can also be detected in human cells, we infected immortalized human foreskin fibroblasts (HFF) with the mixture of the 14 barcoded recombinants. As was observed for the Vero cells, all the barcodes were detected in comparable amounts from the sorted individual cells (Fig 5A). The average number of barcodes detected per cell in HFF was slightly different than observed in Vero cells (lower at MOI 100 and higher at MOI 10; S1 Table). The infected cells were sorted according to their fluorescence: low, intermediate and high (S4B Fig). HFF expressing lower fluorescence replicated a smaller number of viruses than did those expressing intermediate or higher fluorescence (Fig 5B and 5C and S1 Table 1). At both MOIs, differences between low fluorescent cells and cells of either intermediate or high fluorescence were statistically significant (for MOI 10 P < 0.005; for MOI 100 P < 0.001, Fig 5B and 5C, respectively). The difference between the intermediate and high groups was minimal. The number of barcodes per cell from 66 cells infected at MOI 10 and 69 cells infected at MOI 100 were plotted against the level of fluorescence (Fig 5D and 5E). We calculated the Pearson correlation between these parameters for each MOI (0.55 and 0.47 for MOI 10 and 100, respectively, P < 0.001 for both MOIs). Our results show that the correlation between fluorescence level and the NOIVGR can be detected in HFF. We conclude that this correlation is probably not cell type specific and is a more general characteristic of HSV-1 infection.

### Viral fluorescence levels are not positively correlated with cellular fluorescence levels

**Fig 6:**
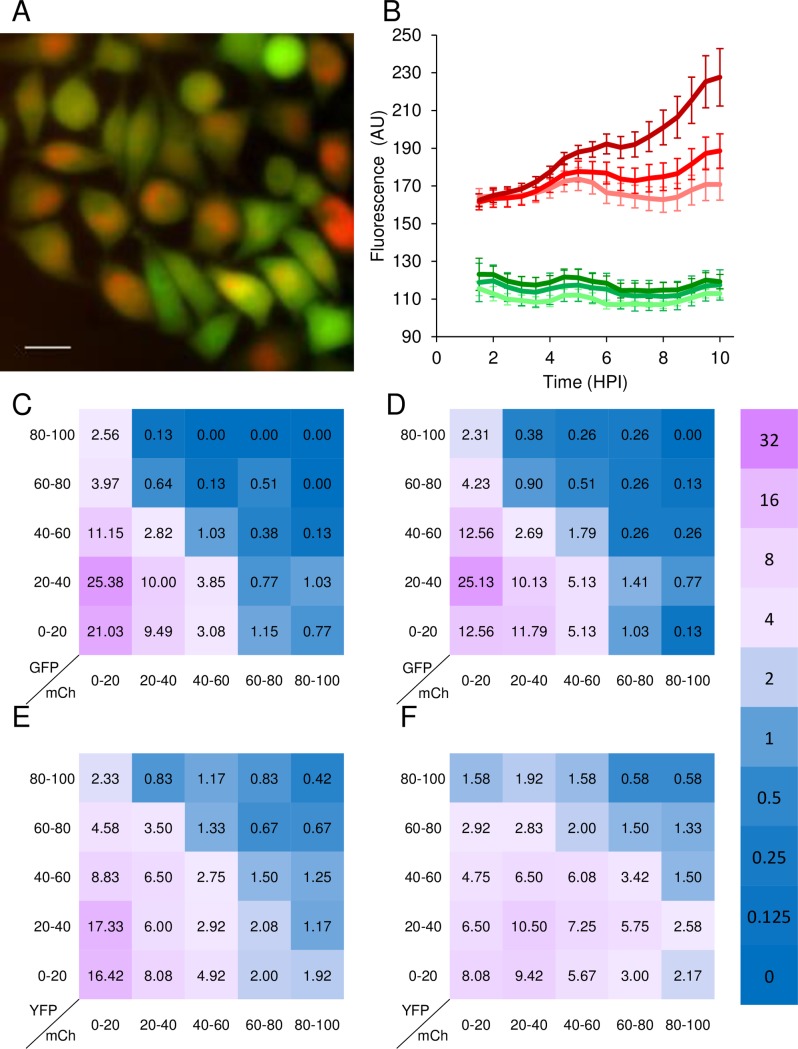
Cells with higher expression of a housekeeping gene have a decreased expression of viral genes. GFP expressing HeLa cells were infected with one of the barcoded viruses. (A). A representative image at 8 HPI is shown. Scale bar 10μm. (B). Fluorescence levels from GFP (shades of green) and mCherry (shade of red) were monitored during HeLa cells infection every 30 minutes for 10 HPI. Infection at MOI 100 (darkest color) or MOI 10 were compared to uninfected cells (lightest color). An average fluorescence of nine frames from cells infected with one of three viral recombinants (three from each recombinant) is shown. Standard deviation for each time point is represented. (C, D) A combination of 780 cells infected with one of three viral recombinants, (two frames from each recombinant, 130 cells per frame) were analyzed from images taken at 10 HPI. Cells were grouped according to the relative fluorescence levels in both GFP and mCherry as indicated for MOI 10 (C) and MOI 100 (D). Each number in the Heatmap represent the proportions of cells out of the total cells analyzed. (E, F) A combination of 1200 cells from 12 different LARCs infected with one viral recombinant, (two frames from each LARC, 50 cells per frame) were analyzed from images taken at 8 HPI. Cells were grouped according to the relative fluorescence levels in both GFP and mCherry as indicated for MOI 10 (E) and MOI 100 (F). Each number in the Heatmap represents the proportions of cells out of the total cells analyzed. The color scheme reflects the relative proportions as indicated in the side bar (log 2 scale).

We speculated that cell to cell variance in viral gene expression (i.e. fluorescence from the viral genomes) depends on the basal gene expression of the specific cell. To test this idea, we compared the level of viral fluorescence to expression from a cellular housekeeping gene (HKG) promoter. HeLa cells harboring the green fluorescent protein (GFP) under phosphoglycerate kinase (PGK) promoter were infected with one of the barcoded viruses. Surprisingly, not only was there no positive correlation between the intensity of the host fluorescence (GFP) with the viral fluorescence (mCherry), (as was shown in Fig 1C and 1D for two viral expressed fluorescent proteins) but in many cases the very highly expressing GFP cells had very little viral expression (Fig 6A). First, we verified that the GFP levels were not affected in the presence of viral infection. Fig 6B shows the average mCherry fluorescence and GFP fluorescence of uninfected green HeLa cells (gHeLa) compared to infected cells at MOI 100 or 10. The results indicate that there was no significant change in the GFP levels during infection. Therefore, the relative levels of GFP expression per cell at the end of experiments represent the relative basal levels of GFP expression prior the infection. We analyzed images at 10 HPI of gHeLa cells infected with one of the barcoded viruses (three different recombinants were used) at MOI 10 or 100. We determine the relative fluorescence (both for mCherry and GFP) for each cell from all eight images and plotted the frequency of the relative fluorescence (combined data from all frames analyzed) as a Heatmap (Fig 6C and 6D for MOI 10 and 100, respectively). Our results indicate that the distribution of the two colors among cells is not random (Chi square test, P<0.005). The proportion of cells with low mCherry levels (left column) with high GFP levels (upper rows) is significantly higher from the expected distribution, in both MOIs (Fig 6C and 6D). Thus, cells with initial high GFP levels are less likely to express high levels of viral gene expression. These results suggest an inverse association between viral gene expression and the basal levels of host HKG expression.

To test whether this association is specific to the promoter tested, we used a set of 12 randomly chosen cell clones from the LARC (library of annotated reporter cell-clones). These cells express the yellow fluorescent protein (YFP) gene fused to different host genes (the specific genes are listed in the methods) and is expressed from the native promoter in its chromosomal position [34]. The 12 LARC were infected with one of the barcoded viruses (each cell line separately). In all cells, the YFP signal was not eliminated during infection F[35]. We analyzed images at 8 HPI following infection at MOI 10 or 100. We accumulated the relative levels of YFP and mCherry from all the cell-lines tested and show the results as Heatmaps for MOI 10 and 100 (Fig 6E and 6F; respectively). Our results indicate that the distribution of the two colors among cells is not random (Chi square test P<0.05). The obtained data show a similar phenomenon as was observed in the gHeLa cells, although it was less distinct, probably because of variability among the LARCs. These results support the inverse association between viral gene expression and the basal levels of host gene expression.

### The number of viral genomes replicated is dependent on cell type

**Fig 7:**
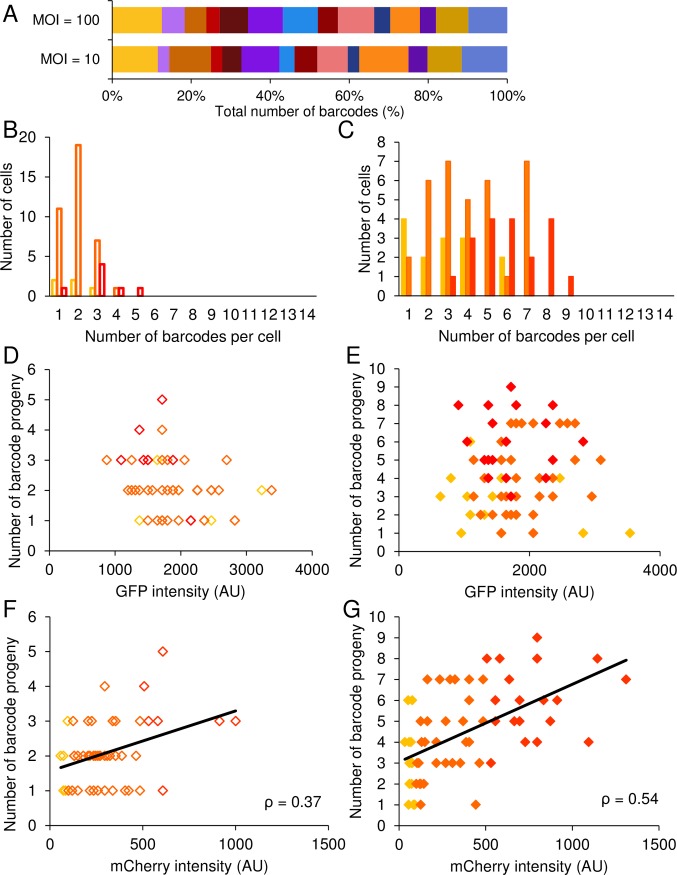
The correlation between the number of barcodes replicating in cells and viral fluorescence is maintained in gHeLa cells. (A) The relative proportion of each barcode of the total number of barcodes detected from all infectious centers analyzed. The results are presented per MOI and each barcode is represented by a different color. (B-G) The output infectious centers, each originated from one individual infected gHela cell, were lysed and analyzed with qPCR. A total of 117 cells from four experiments are presented. For each MOI (10—open bars/diamonds and 100—solid bars/diamonds), the cells are divided according to fluorescence intensity, from low (yellow), through intermediate (orange) and high (red). (B, C) A distribution of the number of barcodes per cell is depicted at MOI 10 (B) and MOI 100 (C). (D, E) The number of replicated barcode progeny from individual cells, infected at MOI 10 (D) and 100 (E), was plotted against expressed green (cellular) fluorescence, as measured by the cell sorter. (F, G) The number of replicated barcode progeny from individual cells, infected at MOI 10 (F) and 100 (G), was plotted against expressed red (viral) fluorescence, as measured by the cell sorter. A trend line that was calculated using the ordinary least squares (OLS) method and the measured Pearson correlation is presented in each graph.

To further study the NOIVGR in different cell types, we repeated the single cell analysis (as described above) on gHeLa cells. We accumulated 50 cells infected at MOI 10, and 67 cells at MOI 100. All fourteen barcodes were detected at each MOI (Fig 7A). The barcode distribution in the gHeLa cells was noisier than in Vero or HFF cells (compare with Figs 4A and 5A). The noisier distribution of barcodes suggests a lower number of barcodes per gHeLa cell. Indeed, we found a lower mean of barcodes per gHeLa cell in both MOI 10 and 100 (2.1 and 4.4, respectively, Fig 7B and 7C and S1 Table) compared with Vero and HFF cells. Single cell HGK expression levels (GFP fluorescence) were not found to correlate significantly with the NOIVGR (Fig 7D and 7E) or with viral gene expression (S6 Fig). We assume that this is due in part to the small sample size and to the small diversification in the GFP levels. We sorted HeLa cells according to their mCherry fluorescence: low, intermediate and high (S4C Fig) and found that the dependence of the NOIVGR on the viral gene expression was maintained in the gHeLa cells. The correlation was clear in MOI 100 (p<0.001, Fig 7G). At MOI 10, the low number of barcodes per cell yielded a result with less statistical significance (p<0.01, Fig 7F). We conclude that while different cell types have different mean NOIVGR per cell; the correlation of this number to viral gene expression levels is likely to be cell type independent, and thus represents a fundamental process during viral infection.

### Cellular parameters can predict the number of barcoded genomes replicated per cell

To assess the predictive value of single cell parameters in estimating the NOIVGR per individual cell, we developed a linear model based on the experiments described above. We extracted a linear relation between the number of barcoded progeny (θ) to MOI and single cell parameters, as measured by the cell-sorter. We used 137 single Vero cells from three independent experiments, 135 single HFF from three independent experiments and 117 single HeLa cells from four independent experiments to calculate the linear equation, using the SPSS linear regression tool. We defined θ as the dependent variable and included MOI, FSC (cell size), SSC (cellular granularity) and mCherry fluorescence as variables for analysis (Eq (1)). The model suggests that the coefficients of MOI, fluorescence, FSC and SSC are significant (P < 0.001, for MOI, fluorescence and FSC; and P < 0.05 for SSC).

θ=-1.102+0.034•MOI+0.004•mChFluorescence+0.012•FSC+0.015•SSC+ε EQUATION (1)

**Fig 8:**
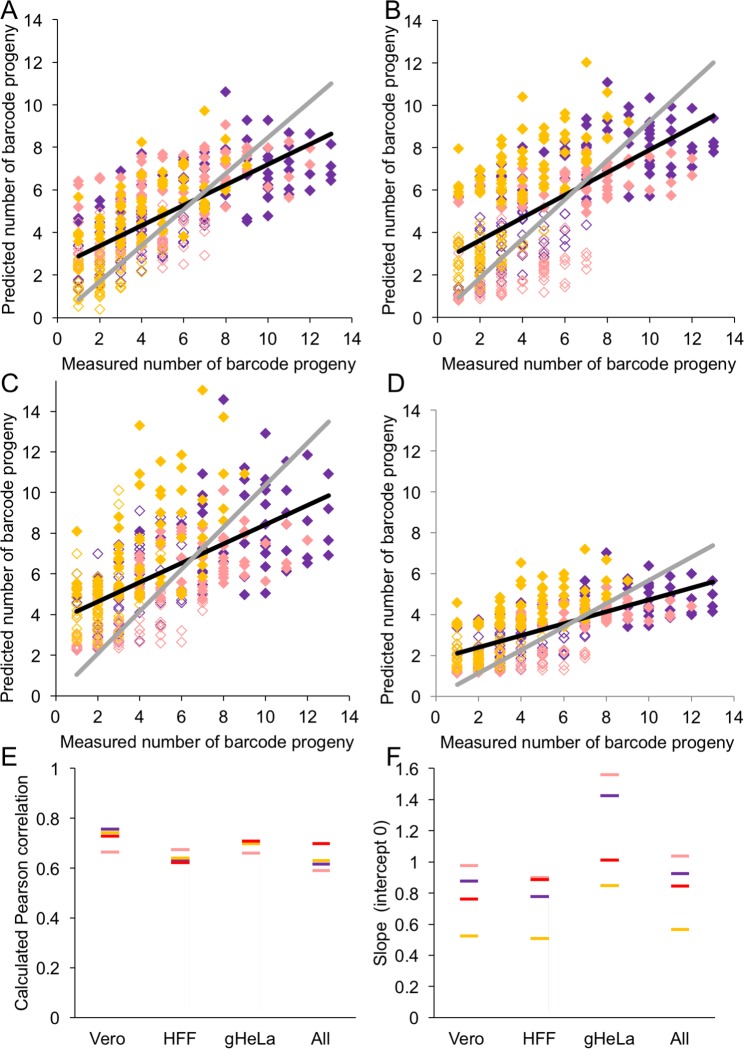
A mathematical model predicts the number of barcodes replicated per cell. Four linear models based on results from all 389 cells (A), Vero cells (B), HFF (C) and gHeLa cells (D) were constructed. The Vero (purple), HFF (light red) and HeLa (orange) cells infected in MOI 10 (open diamonds) and MOI 100 (solid diamonds) are plotted. The predicted number of barcodes per cell was plotted against the measured number of barcodes for all 389 cells. Linear trend lines for each experiment are marked (best fit in black and forced to intercept 0 in gray). (E, F) Results derived from all 389 cells (red), Vero cells (purple), HFF (light red) and gHeLa cells (yellow) are shown. (E) Pearson correlations from each model are plotted against each cell type separately and all together (as indicated). (F) The slopes of the linear trend lines (forced to intercept at 0; slopes of gray lines in A-D and S6 Fig) are plotted.

To test the linear model, we calculated the Pearson correlation between the predicted values obtained from the model and the measured values for each cell. First, all 389 single cells used to develop the model were compared to their predicted values from the model (Fig 8A and S6A, S6E and S6I Fig). The calculated Pearson correlation (0.70, P < 0.001) suggests that while the model predicts the NOIVGR, other single cell parameters should be included to obtain a better estimate. To test the predictive power of this system, we constructed a linear regression model from either of the cell types (each alone) and used these models to asses all cells (Fig 8B–8D and S6B–S6D, S6F–S6H and S6J–S6L Fig). The calculated Pearson correlation from all models on all cell types separately and together are presented in Fig 8E. The calculated correlations obtained from each of the models are comparable (All Pearson correlations were above 0.60, except for the HHF based model predicting all cells which was 0.59). The values of the Pearson correlations indicate that our model can identify the cells that have the highest (or lowest) NOIVGR in any experiment. However, Pearson correlation is not a good indicator for the ability of the model to identify the actual NOIVGR per cell. To test the capability of the model to retrieve the actual NOIVGR, we calculated slopes of the best-fitted trend-lines (forced to intercept at 0) from these models (Fig 8F). We found that the model obtained by either Vero cell experiments or HFF experiments over-estimated the NOIVGR in HeLa cells (slopes ~1.5). Similarly, the model obtained by HeLa cell experiments under-estimated the NOIVGR in Vero cells, HFF and all cells (slopes ~0.5). These differences are probably the result of the differences in the average NOIVGR observed per cell type. Thus, our model can predict NOIVGR in a single cell, according to MOI and single cell parameters determined by the cell sorter. Further, observations of correlations among parameters of different cell types suggest that basic parameters of our models are fundamental to the infection process, and have a predictive property for different cell types.

## Discussion

Cell-to-cell variations during viral infection contribute to significant differences in the outcomes of infection [36–39]. To study sources of single cell diversity during herpesvirus infection, we constructed fourteen barcoded, fluorescence-expressing viral recombinants. The expressed fluorescence from infected cells was found to be a surrogate for viral gene expression. We compared fluorescence levels in individual cells to the number of incoming viral genomes replicating (NOIVGR) in these cells. In the three cell types we examined, the NOIVGR correlated with fluorescence expressed from the viral genome, even though differences in the mean NOIVGR among the cell types were observed. Unexpectedly, our results suggest that cells with high basal gene expression levels are less permissive for viral infection. We also developed multiple linear regression models that predict the NOIVGR in a single cell according to cellular parameters, suggesting that the correlations we observed can be extrapolated to other experimental systems.

We followed the fluorescence level of mCherry expressed from the CMV promoter cloned into the viral genome. We found that fluorescence expressed by cells coincides with viral gene expression throughout the entire infection process (Fig 1). Further, we showed that infected cells maintained their initial relative fluorescence (Fig 2). Taken together, we used the fluorescence level of infected cells at a single time point as an indicator of viral gene expression during infection. We detected small subpopulations of cells in which relative fluorescence changed during infection. Most cells that changed from the highest expression group to the lowest, probably represent cytopathic effect in which the cells lose their focal point and fluorescence (evidence for these cells can be seen in Fig 2B). Cells that changed from the lowest expression group to the highest, are cells in which the fluorescence initiated relatively late, but once initiated, it accumulated fast. This is unlikely to be due to more viral genomes that establish infection and replication at later time points, as no such cells were identified with the three color infections (Fig 2F and 2G and S3 Movie). We therefore speculate that in these cells, a threshold level of viral proteins may be needed to achieve efficient expression and replication, as was previously suggested for reactivation process [40].

To identify factors that influence viral gene expression in an infected cell, we obtained the NOIVGR and cellular parameters from the cell sorter. Fluorescence, as measured by the sorter, was used as a surrogate for viral gene expression. We consistently observed a correlation between fluorescence and the number of barcoded progeny viruses. The number of DNA copies of a gene affects the level of gene expression [41, 42, 43]. In this case, the higher the number of viral genomes initiating expression, the more gene expression (fluorescence) is observed. On the other hand, NOIVGR in a given cell does not necessarily represent the actual viral genome copy number during lytic replication, as genome number probably changes rapidly in an asynchronous manner among cells [26, 28, 44].

The correlation between NOIVGR and gene expression levels (fluorescence) may reflect the initial expression level from the genomes. If viral immediate-early genes are expressed efficiently, they are likely to create conditions that favor expression and replication of co-infecting viral genomes [45–48]. This immediate-early gene hypothesis suggests that viral gene expression may depend on pre-existing single cell determinants. On the other hand, the copy number hypothesis represents a model in which the NOIVGR is stochastic and determines the outcome of infection. These hypotheses are not mutually exclusive and we suggest that both contribute to the outcome of infection in a single cell.

In all the cell types tested, the distribution of the barcode number per cell (Figs 4, 5, and 7) approximates a Poisson distribution for almost any sub-population tested (except for the intermediate HeLa cells at MOI 10). Approximation to the Poisson distribution suggests that each cell contains a different number of viral genomes independent of other cells in the population.

With the 14 barcodes, we can detect up to 14 different parental genomes. However, we cannot exclude the possibility that several parental genomes with the same barcode may enter a single cell. To estimate the difference between the numbers of output barcodes to the number of genomes initiating replication, we calculated the expected values of NOIVGR given the observed number of barcodes (S2 Table). As expected, the overall average number of barcodes detected per cell is lower than the actual NOIVGR. For example, we calculated that for Vero cells at MOI 10, cells with four barcodes have an expected value of 4.5 NOIVGR, meaning that for Vero cells with four barcodes infected at MOI 10, the average number of genomes replicated in these cells is 4.5; as none of the cells can have less than four genomes, at least half of the cells will be replicating four genomes. Our calculations suggest that the barcode system provide a close estimate to the actual number of genomes initiating replication, especially for the cells in which a low number of barcodes was detected per cell. Similarly, at specific conditions, in which the average number of barcodes per cell is lower than other condition (for example: MOI 10 and 100, respectively), the results obtained by the barcodes are closer to the true NOIVGR.

Previously, we found that on average, only a limited number of incoming herpes genomes are expressed in each cell. These results were obtained using three recombinant viruses, each expressing a different fluorescent protein (As in Fig 2F and 2G). Cells infected with an equal mixture of the three recombinant viruses were imaged and analyzed for the presence of fluorescence at each color. A mathematical model predicted that on average less than ten genomes are expressed per cell [29, 31, 32]. In the current work, we measured the number of barcodes in progeny viruses originating from single cells, and used these data to estimate the NOIVGR in the original isolated single cell. It is important to note that in the previous work, we measured only the average numbers of genomes and not the distribution, thus we could not estimate the number of genomes expressed per cell. With the barcode system we observed up to 13 barcodes in one cell, indicating even a higher number of genomes initiating replication (S2 Table). Despite differences between the assays, the results are consistent, indicating that the average number of replicating parental genomes is similar to the average number of expressed parental genomes. Everett and colleagues, and our work, suggested that each RC originates from a single parental genome [28, 29]. However, Phelan *et al*. suggested that the number of RCs is higher than the number of expression foci late after infection [30]. Our data suggest that the RCs observed late during replication by Phelan *et al*. originated from genomes already replicating within the cell.

The combination of single cell analysis with the barcode genetic system provides a new method to study the effect of bottlenecks imposed by single cells on the genetic diversity of DNA viruses. The limited capacity of single cells to replicate multiple RNA viral genomes was recently described as a bottleneck that limits viral diversity. Interestingly, the investigators of that study found that the reduced genetic diversity arising from a single cell correlates with the viral yield from that cell [49]. We suggest that barcode system serves as a measurement of the bottleneck of viral diversity. Similar to the situation with RNA viruses, our results suggest a positive correlation between gene expression of DNA viruses and genetic diversity (barcodes). Thus, single cells are an inherent limiting factor for viral diversity.

To test the effect of cell basal expression levels on viral gene expression, we measured fluorescence obtained from the housekeeping gene PGK promoter. The PGK promoter is commonly used to provide stable expression in mammalian cells [50]. Unexpectedly, basal expression levels did not positively correlate with viral gene expression. Further, cells with high host gene expression were less likely to express high viral expression (Fig 6A–6D). To investigate whether this phenomenon is unique to the PGK promoter, we tested a dozen more host promoters (these promoters are considered active in the cell at all times). The analysis of these twelve promoters together showed a similar phenomenon (Fig 6E and 6F) in which the high host expression levels are less likely to be infected efficiently. Similarly, but less predominant, we observed that cells with high viral gene expression are likely to have low host gene expression. In contrast, two promoters expressed from the same virus are mostly positively correlated (Fig 1C and 1D). In addition, GFP expression from the HSV-1 genome is not inhibitory for viral acute replication [51], suggesting the effect we observe is not due to inhibitory effect of GFP (or its YFP derivative) on viral replication. Taken together, we suggest that cells with higher basal level of host gene expression, also have higher levels of restriction factors (i.e. intrinsic immunity proteins reviewed in [23]), which, by definition, are genes that can be found in the cell at all times. The consequence is a more effective inhibition of viral gene expression. Indeed, a work from our lab suggests that a small increase in known host restriction factors against HSV-1 can result in a decrease in the average number of herpes viral genomes that establish infection per cell [52].

Here we observe differences in the average NOIVGR between the different cell types we tested. While the two immortalized cell types (Vero and HFF) show a similar average number, the cancerous HeLa cells show a significant decrease in the average number of genomes. As HeLa cells genome carries many abnormalities and gene duplications [53], it is possible that some host restriction factors are overexpressed in these cells, which may explain the reduced number of viral genomes expressed. On the other hand, due to genetic abnormalities, HeLa cells may have deleted, mutated or downregulated genes that are required for efficient initiation of viral replication.

All viruses were grown and tittered on Vero cells before they were used in the various experiments. Indeed, this potentially could be a source for differences between cell types, as some of the tegument proteins in the virion originate from the host cell [54]. However, as the average NOIVGR of HFF is similar to the average of Vero, and the efficiency of plating is also similar between all three cell-types, we assume that this experimental parameter has only minimal effect (if any) on the results presented.

We developed a model based on all single cell experiments that estimate the number of parental viral genomes that replicate in any given cell. This model was constructed as a multiple linear regression with all possible explanatory variables (MOI, fluorescence, FSC and SSC). Eq 1 indicates that all measured parameters are positively correlated to the NOIVRG. The effects of MOI and viral fluorescence on the NOIVGR were discussed above. FSC (cell size) and SSC (cell granularity) can distinguish between the different cell types tested. For example, HFF and Vero are larger cells than HeLa (higher FSC, S5 Fig). Taken together, all the parameters allow the prediction of NOIVGR depending on cell type, fluorescence and MOI. The model predicted the results of all cells together, as well as each cell-type separately (Fig 8 and S7 Fig). We developed similar models based on results obtained from only one of the three cell-types. Remarkably, these models had similar Pearson correlation values for each experiment, as did the original model, although a bias was observed among the cell-types, which corresponded to the difference in mean NOIVGR measured. We conclude that the correlation between the NOIVGR and the cellular parameters measured is a robust phenomenon and may be extrapolated to different cell-types.

In conclusion, we developed an experimental system that identifies single cell variation during HSV-1 infection. Further, this system provides evidence to the connection between viral gene expression and viral diversity. We suggest that the high basal level of gene expression in a given cell is a key determinant for reducing the probability for initiation of infection from herpes genomes. Our results support the hypothesis that host cell determinants, in combination with the stochastic nature of viral infection, contribute to the outcome of infection.

## Methods

### Cells

The single cell experiments were performed with either green monkey kidney cells (Vero cells, ATCC CCL-81), immortalized human foreskin fibroblasts (HFF) or human cervical cancer cells (HeLa cells). These HFF cells were immortalized by hTERT transfection. The immortalized HFF cells were a kind gift from the Sara Selig. HeLa cells used in this work were infected with a lentivirus carrying the GFP gene under the mouse PGK promoter, derived from the pMSCVpuro plasmid (Clontech). These HeLa cells were a kind gift from the E. Bacharach lab. The HeLa cells were sorted into single cells to ensure all experiments were done from a single clone, and that GFP expression differences are not due to insertion site. Cells were grown with Dulbecco's Modified Eagle Medium (DMEM X1; Gibco), supplemented with 10% Fetal Calf Serum (FBS; Gibco) and 1% penicillin (10,000 units/ml) and Streptomycin (10mg/ml) (Biological Industries Israel) at 37°C and 5% CO2. The HeLa cells were grown in the presence of G418 (0.5mg/ml Gibco) to maintain fluorescence expression.

12 randomly chosen clones from the LARC (library of annotated reporter cell-clones) were a kind gift from Uri Alon. These cells originated from the non-small cell lung carcinoma cell-line, H1299. Each of the clones has the yellow fluorescent protein (YFP) gene fused to different host gene and is expressed from the native promoter in its chromosomal position [34]. The 12 clones used in this study are: NASP, DNMT1, SUMO1, CIRBP, LMNA, LMNB1, ACTN4, H2AFV, FBL, RPS11, MSN and TOP1. Cells were grown in RPMI supplemented with 10% FBS (Gibco) and 1% penicillin (10,000 units/ml) and Streptomycin (10mg/ml) (Biological Industries Israel). at 37°C and 8% CO2.

### Viruses

All viruses are derivatives of HSV-1 strain17+. Viral recombinants OK11, OK12 and OK22 carry a single fluorescent protein (mCherry, EYFP and mTurq2, respectively) with a nuclear localization tag under the CMV promoter between UL37 and UL38 genes as described previously [32, 33]. Viral recombinant OK21 was created by a cross between OK11 and OK19 (a HSV-1 strain17+ containing the mVenus gene fused in frame within the UL25 gene after the 50^th^ amino acid of the viral protein similar to what was previously described [55]).

### Barcode virus system construction

Fourteen 16-nucleotide sequences were randomly generated, and the sequence of BclI restriction site (GATC) was added to each. The resulting fourteen 20bp sequences were synthesized by Integrated DNA Technologies, Inc. (IDT) and cloned into pOK11 plasmids (an mCherry with a triplicate nuclear localization sequence (3xNLS) under the CMV Promoter in between homology regions of HSV-1 UL37 and UL38 genes) as was previously described [32]. The 14 generated plasmids were cotransfected (TRANSFECTENE@ PRO, Biontex) into Vero cells with purified HSV-1 strain 17 DNA [56]. Each recombinant was plaque purified according to fluorescence and analyzed with qPCR to verify the insertion. The growth properties of each recombinant were compared to the parental strain, using a single-step growth curve analysis (S1 Fig). All viral stocks were cultured and tittered on Vero cells.

### Single cell sorting

Vero, HFF or gHeLa cells were infected with an equal mixture of barcoded viruses at different MOIs, as specified in each experiment. At 3 HPI, the cells were trypsinized and kept on ice until sorting. The cells were sorted with Astrios (Beckman-Coulter) and distributed one cell per well on a 96-well plate, pre-seeded with uninfected Vero cells. To ensure reproducibility between the experiments, each experiment uninfected cells were sorted first, and the average and median fluorescence were kept at ~30 fluorescence arbitrary units (AU). The fluorescence cut-offs for these experiments were set to 100 (low to intermediate) and 500 (intermediate to high) AU for Vero and gHeLa. HFF expressed lower fluorescence levels, thus the cut-offs for these cells were set to 100 and 350 AU. To verify that only one cell was placed in each well, the 96-well plate was scanned under a fluorescence microscope (Nikon Eclipse Ti-E) at 24 and 48 HPI to verify that only one infectious center was observed in each well. When an infected cell was detected at 24 HPI, but did not develop into an infectious center, we determined it as an abortive infection. We conducted three experiments in Vero cells, three experiments in HFF and four in HeLa cells; all experiments were performed in MOI 10 and 100. For Vero cells, 200 single cells were identified, of which 142 were further analyzed. Ten wells (~2%) contained more than one plaque focus. For HFF, 192 single cells were identified and 141 were further analyzed with qPCR. 19 wells (~3%) contained plaques with at least two foci. For HeLa cells, 124 productive single cells were identified and analyzed. In addition to these cells, 61 infected single cells were identified that did not develop into infectious centers. Fourteen percent of the wells contained at least two focus plaques.

### Barcode quantitative PCR (qPCR)

Wells with one infectious center were incubated for 6 dpi (days post infection), and then incubated for one hour with lysis buffer containing 10mM Tris-HCl, pH = 8.0, 1mM EDTA (Merck), 1% Tween 20 (Sigma-Aldrich), 0.04% Proteinase K (BIO-LAB, Israel) [57]. Proteinase K was deactivated by exposure to 95°C for 10 minutes. The lysate was kept at 37°C for an additional 24 hours, prior to analysis with qPCR. The samples were analyzed with qPCR (QuantStudio 12K Flex, Applied Biosystems), using SYBR master mix (Applied Biosystems). A list of primers used for the qPCR assay is specified in S3 Table.

### Data and statistical analysis

Parameters of each analyzed single cell were collected during sorting with Summit 6.2 (Beckman-Coulter). These included FSC, SSC and fluorescence (561/14). Further analysis measured the number of barcodes for each single cell. The Thompson tau method was used as an initial analysis to discover fluorescence/barcode outliers. Of 142 Vero, 141 HFF and 124 gHeLa single cells that were analyzed, four Vero, three HFF and five gHeLa single cells in MOI 10; and one Vero, three HFF and two gHeLa single cells in MOI 100 were outliers. After their removal, 137 Vero, 135 HFF and 117 gHeLa single cells were used for further analysis.

We performed student's t-test and one-way ANOVA statistical tests and calculated Pearson correlation with SPSS statistical software (IBM). To test whether Poisson distribution was a good approximation for number of barcodes per cell distribution, we used the Chi-Square Goodness of fit test. Since only infected cells where examined, we considered distribution of positive numbers, thus excluding the option of zero (i.e. uninfected cells). This required an adjustment of the Poisson distribution with Maximum Likelihood estimate.

### Live cell imaging

For testing viral gene expression maintenance throughout the infection, Vero cells were infected with an equal mixture of 14 barcoded recombinants in MOI 10 or 100. Infections were monitored every 10 minutes for 18 hours. Four frames per well and three wells per MOI were visualized. For testing color change throughout the infection, Vero cells were infected with an equal mixture of 3 color recombinants in MOI 100. Infections were monitored every 15 minutes for 16 hours. All the movies were taken using a Nikon Eclipse Ti-E epifluorescence inverted microscope with a heated stage top incubator (Chamlide, live cell instrument).

### Image analysis

The movies were analyzed with Imaris software (Bitplane) to discover the increase in mCherry fluorescence. Data were normalized by a moving average of five time points for each cell. We note that for each movie, several images showed a dramatic decrease in the fluorescence level for both channels. This anomaly reflected random electrical power problems. To overcome this, we used an average of time points before and after the decrease as an estimate for fluorescence level.

### RT-qPCR

Five different experiments were performed on different days, for each experiment from 600,000 to 1,150,000 cells per population were taken (population size was equal within each experiment). Cells infected with an MOI 10 or 100 were trypsinized at 3 HPI and centrifuged at 1,500 rpm (264g) in 4°C for 2 minutes. Cells were resuspended in FACS buffer (Dulbacco's phosphate buffered saline PBSX1, 5mM EDTA, 1% FBS). Cells were sorted with Astrios (Beckman-Coulter) into three populations according to their fluorescence (lowest, middle, highest). After sorting, cells were centrifuged in 1,500rpm in 4°C for 10 minutes. After the removal of the supernatant, Bio-Tri RNA Reagent (BIO-LAB, Israel) was added according to the number of sorted cells and immediately frozen in -80°C. The RNA was produced following the manufacturer protocol, with materials adjusted to the number of sorted cells and immediately frozen in -80°C. The RNA samples were quantified with Nanodrop (Thermo Scientific) and quality of the RNA sample was determined. Equal amounts of RNA (per experiment) were taken to produce cDNA. cDNA was produced, using random primers, with RevertAid first strand cDNA kit (Thermo scientific) or High-Capacity cDNA Reverse Transcription Kit (Applied Biosystems) and the resulting cDNA was used for qPCR analysis (QuantStudio 12K Flex, Applied Biosystems), using SYBR master mix (Applied Biosystems). Each cDNA sample was analyzed with viral immediate-early, early, late genes, and a cellular housekeeping gene (HMBS). The sequences for primers for viral genes and cellular housekeeping gene appear in S4 Table.

## Supporting Information

S1 Fig Single-step growth curve of barcoded virus recombinants. Vero cells were infected at MOI of 10 with one of the barcoded virus recombinants (shades of gray) and compared to the wild type virus (red). Each point is an average of viral titers obtained from three technical replicates. Error bars show standard error.
(PDF)

S2 Fig Each barcoded recombinant can be detected by quantitative PCR with a selective primer. Viral lysates of the 14 barcoded recombinants were categorized into 3 groups according to their genetic differences (using the Clustal Omega online tool for multiple sequence alignment and phylogeny tree). In each group the lysates were tested against the specific primer of each group member. Further, a mixture of the lysates from that group was tested against a mixture of primers from the two other groups. Amplification is noted with the + sign (lower than 25 PCR cycles) and no product is presented with the–sign (more than 35 PCR cycles).
(PDF)

S3 Fig Relative fluorescence of individual cells is maintained throughout infection. Cells infected with a mixture of the barcoded viruses at a MOI of 10 were monitored for 18 hours. (A) Snapshots of cells at different time points (as indicated) are presented. Scale bar 20μm. (B) The fluorescence profile of 98 individual cells from a single well (4 different frames) is presented as a function of time. The profiles were sorted according to the fluorescence intensity of the cells at 4hpi, from low (blue 30% of total), through intermediate (purple 40%) and high (red 30%). (C) The mean of the cell fluorescence profiles for each of the groups of fluorescence intensity was calculated for each well. An average of three wells (with the standard deviation between the wells-stripe lines) is presented (color coded as B). (D, E) At each time point indicated, the cell profiles were sorted according to fluorescence levels. The 30% low population (D) and 30% high population (E) were compared to the 4hpi time point. Each column represents the ratio of cells from the 4hpi segregation as found in the indicated time point. An average of the ratios from three different wells is presented.
(PDF)

S4 Fig Flow cytometry of barcode recombinant infected cells. Populations of cells infected with the mixture of barcoded recombinants at MOI of 10 (green) or 100 (blue) or uninfected cells (red) are plotted according to their fluorescence. Populations collected from three Vero (A), three HFF (B) and four HeLa (C) single-cell sorting experiments are presented. Analysis according to low (L) intermediate (I) and high (H) fluorescent groups is also indicated.
(PDF)

S5 Fig Forward and side scatter are weak predictors for the number of barcodes detected per cell. The number of replicated barcode progeny from individual cells, infected at MOI 10 (A, C, E, G, I and K) and MOI 100 (B, D, F, H, J AND L), was plotted against forward scatter (FSC; A-B, E-F and I-J) and side scatter (SSC; C-D, G-H and K-L) as measured by the cell sorter. Each cell type is color coded according to fluorescence levels expressed from individual cells: Vero cells (A-D) are colored blue for low fluorescence, purple for intermediate fluorescence and red for high fluorescence; HFF (E-H) are colored pink for low fluorescence, light red for intermediate fluorescence and red for high fluorescence; and HeLa cells (I-L) are colored yellow for low fluorescence, orange for intermediate fluorescence and red for high fluorescence. A trend line that was calculated using the ordinary least squares (OLS) method is presented in each graph.
(PDF)

S6 Fig Single cell HGK expression levels does not correlate with viral gene expression. gHeLa cells were infected at MOI 10 (A) or MOI 100 (B) with a mixture of the barcoded viruses. For each cell, the expressed red (viral) fluorescence levels were plotted against the expressed green (cellular) fluorescence levels, as measured by the cell sorter. A trend line that was calculated using the ordinary least squares (OLS) method is presented in each graph.
(PDF)

S7 Fig Four mathematical models predict the number of barcodes replicated per cell. Four different models based on results from all 389 cells (A, E and I), 137 Vero cells (B, F and J), 135 HFF (C, G and K) or 117 HeLa cells (DHL) were constructed. The predicted number of barcodes per cell by each model was compared against the measured number of barcodes for each of the cell types: Vero cells (A-D, purple), HFF (E-H, light red) and HeLa Cell (I-L, yellow) and plotted as indicated. Cells infected in MOI 10 (open diamonds) and MOI 100 (solid diamonds) are plotted. Linear trend lines for each experiment are marked (best fit in black and forced to intercept 0 in gray).
(PDF)

S1 Table Barcode averages and their ratios in the overall population. Means and standard deviations (Std) of the numbers of barcodes per cell are depicted at an MOI of 10 and an MOI of 100, according to cell fluorescence level. The proportions of cells in each group of the total number of cells, as measured by the sorter, are also indicated. A total mean of all the cells, as measured from the experiment (expe) and as calculated (calc) according to the relative proportion of each group, are presented.
(DOCX)

S2 TableThe expected value for the number of replicating viral genomes given the average number of barcodes observed at different experimental conditions.For each condition (columns), we calculated the expected value (*E*(*Y*|*ζ*)) of the number of replicating genomes (Y), assuming *ζ* different barcodes are detected in a single cell. Given the observed average number of barcodes ($\hat{\lambda }$) and the total number of input barcodes (N = 14), the expected value was calculated following the equation:
$$E(Y|\zeta )=\zeta ∙(\frac{\frac{\hat{\lambda }}{N}}{1-e^{-\frac{\hat{\lambda }}{N}}})$$
For each condition we highlighted the most relevant expected values which are closest to the average number of barcodes observed per condition. Experimental data of average and standard deviation of the number of barcodes observed in each condition are listed in the last two rows.(DOCX)

S3 Table List of primer sequences used for barcode analysis. Fourteen sequences that were inserted into wild type HSV-1 genome are presented. These sequences are also the primers with which these viruses are identified with qPCR.
(DOCX)

S4 Table List of primer sequences used for gene expression analysis. Sequences for qPCR primers for six viral genes are given, (two from each gene group: immediate-early, early and late genes). A sequence for a cellular housekeeping gene HMBS is given last.
(DOCX)

S1 Movie Cells infected with a mixture of the barcoded viruses at MOI of 100 were monitored for 18 hours. Cells were treated with NucBlue (molecular probes, USA) according to manufacturer's recommendation, 15min prior to the first image. A representative frame is shown for each MOI. Scale bar 20μM.
(MOV)

S2 Movie Cells infected with a mixture of the barcoded viruses at MOI of 10 were monitored for 18 hours. Cells were treated with NucBlue (molecular probes, USA) according to manufacturer's recommendation, 15min prior to the first image. A representative frame is shown for each MOI. Scale bar 20μM.
(MOV)

S3 Movie Cells were infected with three different fluorescence expressing viral recombinants (each expressing one of the following fluorescent genes: mCherry, EYFP or mTurq2) at MOI of 100. The cells were monitored for 16 hours, every 15 minutes. Scale bar 20μM.
(MOV)
