# Supplementary figures and images for: Gene Expression Correlates with the Number of Herpes Viral Genomes Initiating Infection in Single Cells
Source: PLoS Pathog. 2016 Dec 6;12(12):e1006082. doi: 10.1371/journal.ppat.1006082 (PMC5161387; doi:10.1371/journal.ppat.1006082)

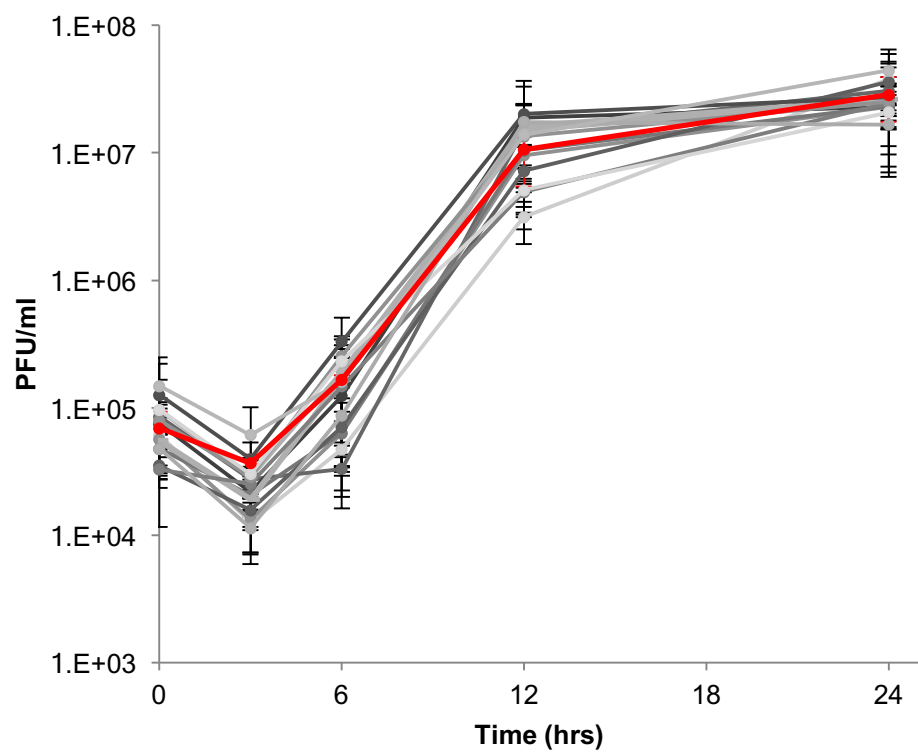

Supplement: S1 Fig — Vero cells were infected at MOI of 10 with one of the barcoded virus recombinants (shades of gray) and compared to the wild type virus (red). Each point is an average of viral titers obtained from three technical replicates. Error bars show standard error. (PDF) [file ppat.1006082.s001.pdf]

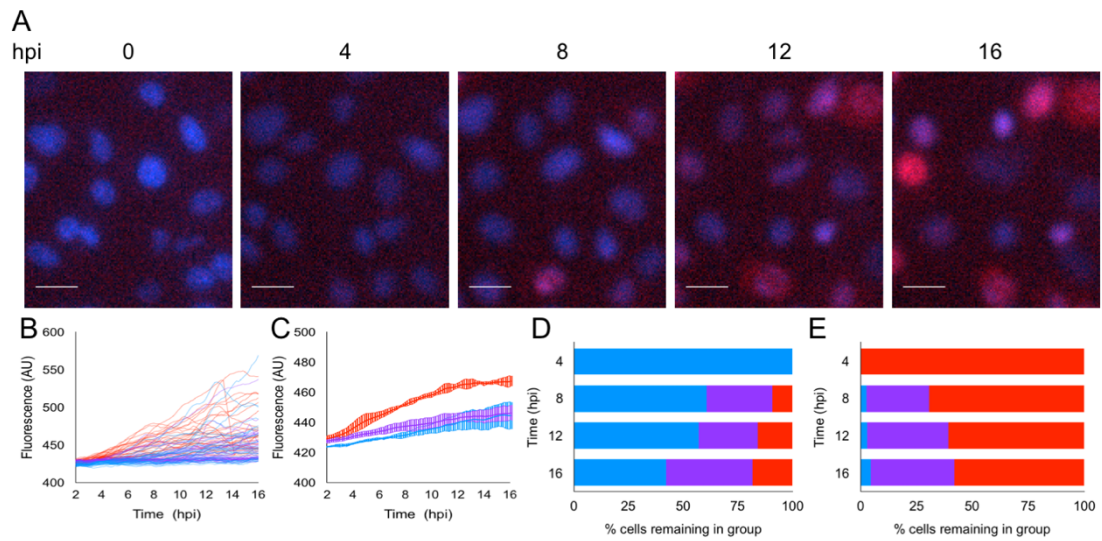

Supplement: S3 Fig — Cells infected with a mixture of the barcoded viruses at a MOI of 10 were monitored for 18 hours. (A) Snapshots of cells at different time points (as indicated) are presented. Scale bar 20μm. (B) The fluorescence profile of 98 individual cells from a single well (4 different frames) is presented as a function of time. The profiles were sorted according to the fluorescence intensity of the cells at 4hpi, from low (blue 30% of total), through intermediate (purple 40%) and high (red 30%). (C) The mean of the cell fluorescence profiles for each of the groups of fluorescence intensity was calculated for each well. An average of three wells (with the standard deviation between the wells-stripe lines) is presented (color coded as B). (D, E) At each time point indicated, the cell profiles were sorted according to fluorescence levels. The 30% low population (D) and 30% high population (E) were compared to the 4hpi time point. Each column represents the ratio of cells from the 4hpi segregation as found in the indicated time point. An average of the ratios from three different wells is presented. (PDF) [file ppat.1006082.s003.pdf]

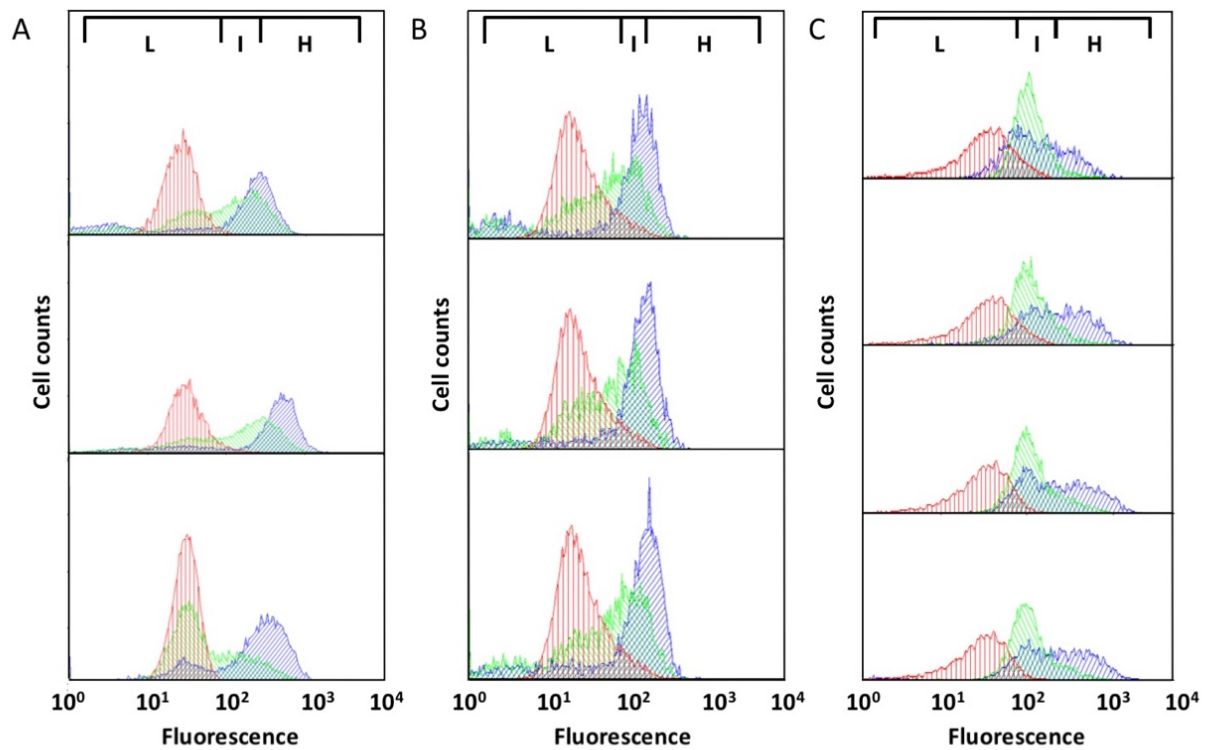

Supplement: S4 Fig — Populations of cells infected with the mixture of barcoded recombinants at MOI of 10 (green) or 100 (blue) or uninfected cells (red) are plotted according to their fluorescence. Populations collected from three Vero (A), three HFF (B) and four HeLa (C) single-cell sorting experiments are presented. Analysis according to low (L) intermediate (I) and high (H) fluorescent groups is also indicated. (PDF) [file ppat.1006082.s004.pdf]

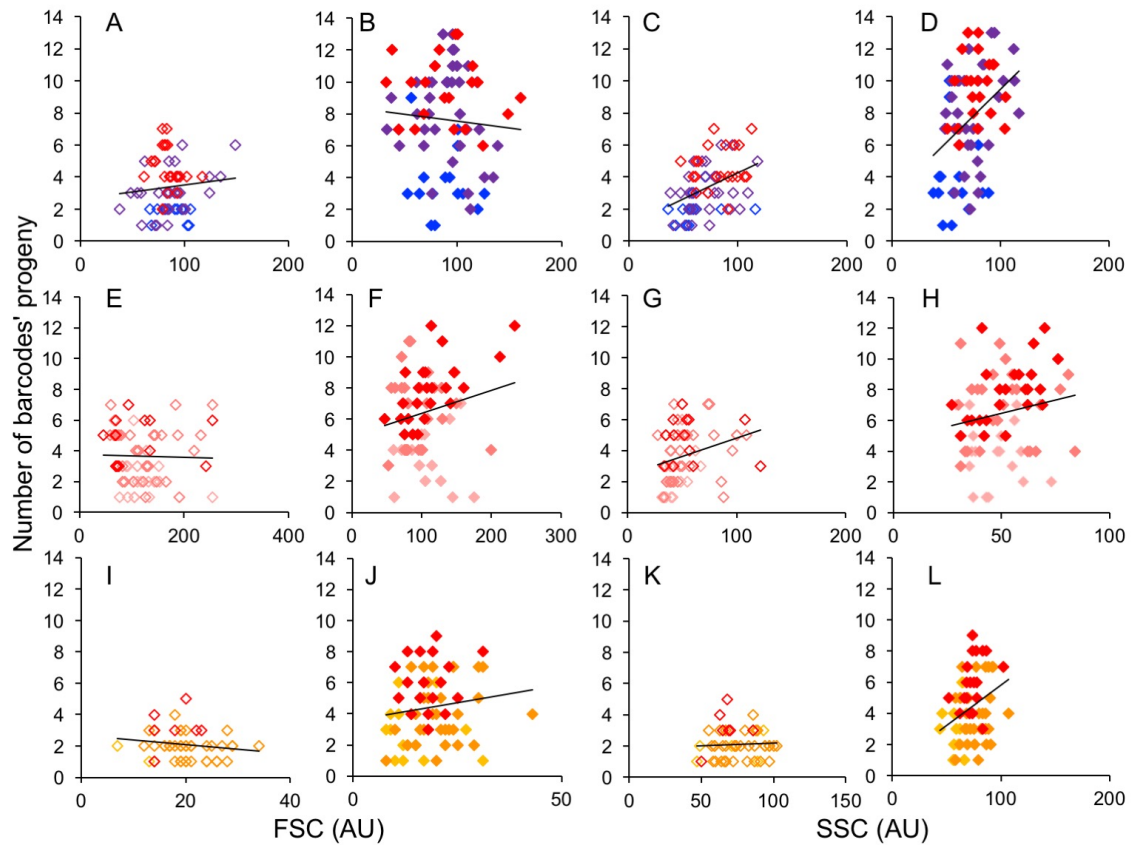

Supplement: S5 Fig — The number of replicated barcode progeny from individual cells, infected at MOI 10 (A, C, E, G, I and K) and MOI 100 (B, D, F, H, J AND L), was plotted against forward scatter (FSC; A-B, E-F and I-J) and side scatter (SSC; C-D, G-H and K-L) as measured by the cell sorter. Each cell type is color coded according to fluorescence levels expressed from individual cells: Vero cells (A-D) are colored blue for low fluorescence, purple for intermediate fluorescence and red for high fluorescence; HFF (E-H) are colored pink for low fluorescence, light red for intermediate fluorescence and red for high fluorescence; and HeLa cells (I-L) are colored yellow for low fluorescence, orange for intermediate fluorescence and red for high fluorescence. A trend line that was calculated using the ordinary least squares (OLS) method is presented in each graph. (PDF) [file ppat.1006082.s005.pdf]

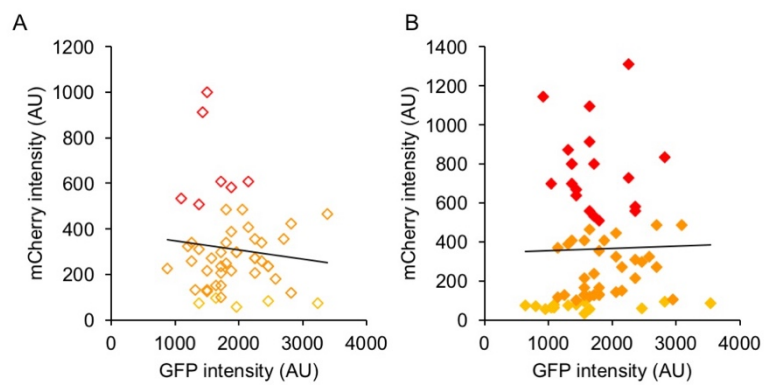

Supplement: S6 Fig — gHeLa cells were infected at MOI 10 (A) or MOI 100 (B) with a mixture of the barcoded viruses. For each cell, the expressed red (viral) fluorescence levels were plotted against the expressed green (cellular) fluorescence levels, as measured by the cell sorter. A trend line that was calculated using the ordinary least squares (OLS) method is presented in each graph. (PDF) [file ppat.1006082.s006.pdf]

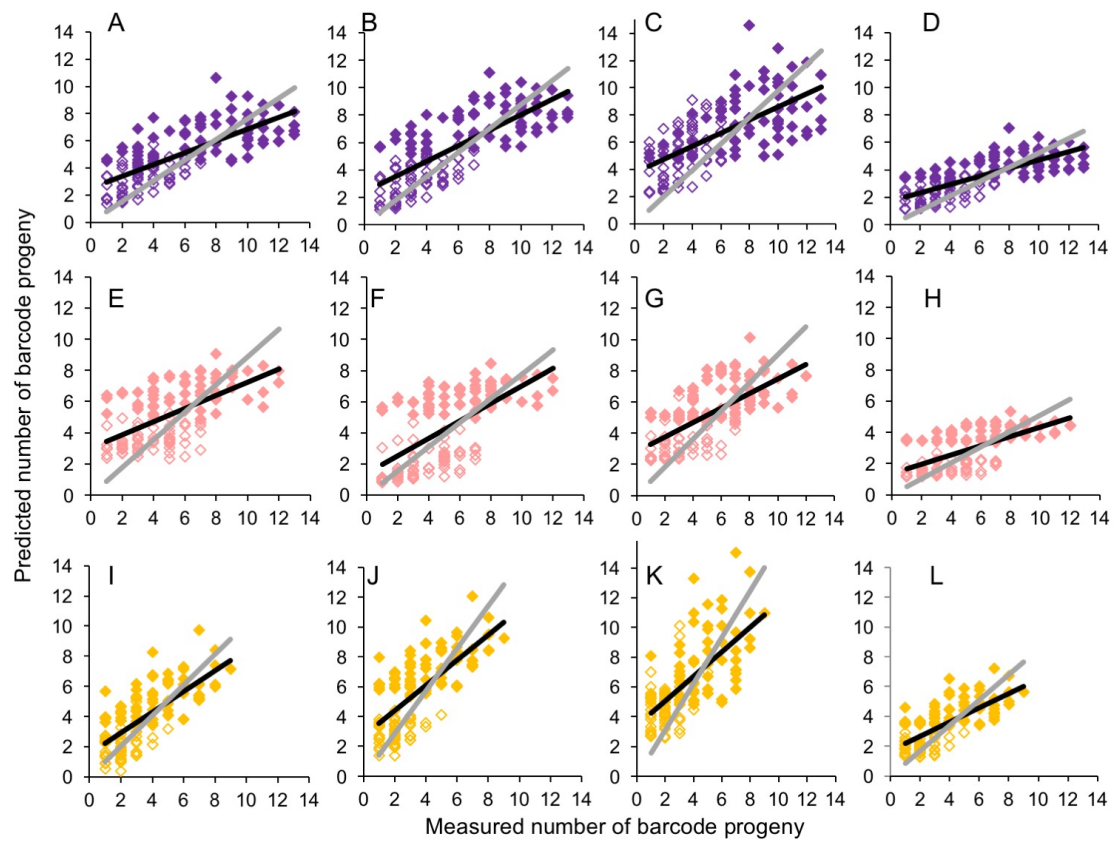

Supplement: S7 Fig — Four different models based on results from all 389 cells (A, E and I), 137 Vero cells (B, F and J), 135 HFF (C, G and K) or 117 HeLa cells (DHL) were constructed. The predicted number of barcodes per cell by each model was compared against the measured number of barcodes for each of the cell types: Vero cells (A-D, purple), HFF (E-H, light red) and HeLa Cell (I-L, yellow) and plotted as indicated. Cells infected in MOI 10 (open diamonds) and MOI 100 (solid diamonds) are plotted. Linear trend lines for each experiment are marked (best fit in black and forced to intercept 0 in gray). (PDF) [file ppat.1006082.s007.pdf]
