# Supplementary material for: Gene Expression Correlates with the Number of Herpes Viral Genomes Initiating Infection in Single Cells
Source: PLoS Pathog. 2016 Dec 6;12(12):e1006082. doi: 10.1371/journal.ppat.1006082 (PMC5161387; doi:10.1371/journal.ppat.1006082)
Supplement: S2 Fig — Viral lysates of the 14 barcoded recombinants were categorized into 3 groups according to their genetic differences (using the Clustal Omega online tool for multiple sequence alignment and phylogeny tree). In each group the lysates were tested against the specific primer of each group member. Further, a mixture of the lysates from that group was tested against a mixture of primers from the two other groups. Amplification is noted with the + sign (lower than 25 PCR cycles) and no product is presented with the–sign (more than 35 PCR cycles). (PDF) [file ppat.1006082.s002.pdf]

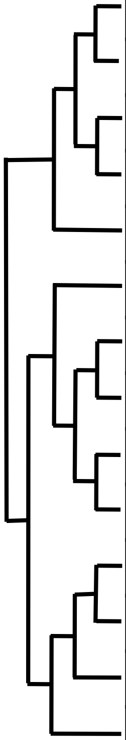

	8	12	6	2	4	11	19	3	15	13	7	23	9	1
8	+	-	-	-	-									
12	-	+	-	-	-									
6	-	-	+	-	-			-				-		
2	-	-	-	+	-									
4	-	-	-	-	+									
11						+	-	-	-	-				
19						-	+	-	-	-				
3			-			-	-	+	-	-		-		
15						-	-	-	+	-				
13						-	-	-	-	+				
7											+	-	-	-
23											-	+	-	-
9			-					-			-	-	+	-
1											-	-	-	+
