# Supplementary material for: Gene Expression Correlates with the Number of Herpes Viral Genomes Initiating Infection in Single Cells
Source: PLoS Pathog. 2016 Dec 6;12(12):e1006082. doi: 10.1371/journal.ppat.1006082 (PMC5161387; doi:10.1371/journal.ppat.1006082)
Supplement: S1 Table — Means and standard deviations (Std) of the numbers of barcodes per cell are depicted at an MOI of 10 and an MOI of 100, according to cell fluorescence level. The proportions of cells in each group of the total number of cells, as measured by the sorter, are also indicated. A total mean of all the cells, as measured from the experiment (expe) and as calculated (calc) according to the relative proportion of each group, are presented. (DOCX) [file ppat.1006082.s008.docx]

|  | **MOI 10** | | | **MOI 100** | | |
| --- | --- | --- | --- | --- | --- | --- |
|  | Mean | Std. | % Population | Mean | Std. | % Population |
| **Vero** |  |  |  |  |  |  |
| Low | 2.00 | 0.82 | 41.04 | 4.13 | 2.60 | 10.42 |
| Intermediate | 3.13 | 1.43 | 38.64 | 8.06 | 2.91 | 32.37 |
| High | 4.62 | 1.32 | 15.50 | 9.55 | 2.04 | 51.79 |
| Total (calc) | 2.75 | 1.16 | 95.18 | 7.99 | 2.37 | 94.58 |
| Total (expe) | 3.39 | 1.60 |  | 7.64 | 3.25 |  |
| **HFF** |  |  |  |  |  |  |
| Low | 2.40 | 1.39 | 66.76 | 3.89 | 2.03 | 18.19 |
| Intermediate | 4.00 | 1.68 | 33.12 | 6.93 | 2.34 | 80.71 |
| High | 4.77 | 1.42 | 0.12 | 7.83 | 2.04 | 1.09 |
| Total (calc) | 2.93 | 1.49 | 99.99 | 6.38 | 2.28 | 99.98 |
| Total (expe) | 3.67 | 1.77 |  | 6.45 | 2.65 |  |
| **HeLa** |  |  |  |  |  |  |
| Low | 1.80 | 0.84 | 39.53 | 2.93 | 1.73 | 22.85 |
| Intermediate | 1.95 | 0.77 | 53.95 | 4.12 | 1.92 | 55.43 |
| High | 3.14 | 1.21 | 2.21 | 6.00 | 1.70 | 19.38 |
| Total (calc) | 1.83 | 0.79 | 95.69 | 4.12 | 1.81 | 97.65 |
| Total (expe) | 2.1 | 0.93 |  | 4.40 | 2.11 |  |
