# Supplementary material for: Gene Expression Correlates with the Number of Herpes Viral Genomes Initiating Infection in Single Cells
Source: PLoS Pathog. 2016 Dec 6;12(12):e1006082. doi: 10.1371/journal.ppat.1006082 (PMC5161387; doi:10.1371/journal.ppat.1006082)
Supplement: S2 Table — For each condition (columns), we calculated the expected value (E(Y|ζ)) of the number of replicating genomes (Y), assuming ζ different barcodes are detected in a single cell. Given the observed average number of barcodes (λ^) and the total number of input barcodes (N = 14), the expected value was calculated following the equation: E(Y|ζ)=ζ∙(λ^N1−e−λ^N) For each condition we highlighted the most relevant expected values which are closest to the average number of barcodes observed per condition. Experimental data of average and standard deviation of the number of barcodes observed in each condition are listed in the last two rows. (DOCX) [file ppat.1006082.s009.docx]

| $\boldsymbol{\zeta}$**- # of barcodes detected per cell** | **VERO** | | **HFF** | | **gHeLa** | |
| --- | --- | --- | --- | --- | --- | --- |
|  | **10** | **100** | **10** | **100** | **10** | **100** |
| **1** | 1.13 | 1.30 | 1.14 | 1.25 | 1.08 | 1.17 |
| **2** | 2.25 | 2.60 | 2.27 | 2.50 | 2.15 | 2.33 |
| **3** | 3.38 | 3.89 | 3.41 | 3.74 | 3.23 | 3.50 |
| **4** | 4.50 | 5.19 | 4.55 | 4.99 | 4.31 | 4.66 |
| **5** | 5.63 | 6.49 | 5.68 | 6.24 | 5.38 | 5.83 |
| **6** | 6.76 | 7.79 | 6.82 | 7.49 | 6.46 | 6.99 |
| **7** | 7.88 | 9.09 | 7.96 | 8.74 | 7.54 | 8.16 |
| **8** | 9.01 | 10.38 | 9.09 | 9.98 | 8.61 | 9.32 |
| **9** | 10.13 | 11.68 | 10.23 | 11.23 | 9.69 | 10.49 |
| **10** | 11.26 | 12.98 | 11.37 | 12.48 | 10.77 | 11.65 |
| **11** | 12.39 | 14.27 | 12.50 | 13.73 | 11.85 | 12.82 |
| **12** | 13.51 | 15.57 | 13.64 | 14.98 | 12.92 | 13.98 |
| **13** | 14.64 | 16.87 | 14.78 | 16.22 | 14.00 | 15.15 |
| **14** | 15.76 | 18.17 | 15.92 | 17.47 | 15.08 | 16.32 |
| Experimental Avg. | 3.39 | 7.64 | 3.67 | 6.45 | 2.1 | 4.4 |
| Experimental Std. | 1.6 | 3.25 | 1.77 | 2.65 | 0.93 | 2.11 |
