# Supplementary material for: Gene Expression Correlates with the Number of Herpes Viral Genomes Initiating Infection in Single Cells
Source: PLoS Pathog. 2016 Dec 6;12(12):e1006082. doi: 10.1371/journal.ppat.1006082 (PMC5161387; doi:10.1371/journal.ppat.1006082)
Supplement: S3 Table — Fourteen sequences that were inserted into wild type HSV-1 genome are presented. These sequences are also the primers with which these viruses are identified with qPCR. (DOCX) [file ppat.1006082.s010.docx]

| **Primer** | **Sequence (5' -> 3')** |
| --- | --- |
| mCherry (forward) | GCATGGACGAGCTGTACAAG |
| BC1 | GATCGTACCCACACCACATG |
| BC2 | GATCCATGTGGTGTGGGTAC |
| BC3 | GATCGTTGCACGTCCAGATC |
| BC4 | GATCGATCTGGACGTGCAAC |
| BC6 | GATCCATGACGAGAGCGAAC |
| BC7 | GATCGTACCGTTGCGTGTTG |
| BC8 | GATCCAACACGCAACGGTAC |
| BC9 | GATCGTACGGACAGGTCTTC |
| BC11 | GATCGTTGCGAGAGGAGTTG |
| BC12 | GATCCAACTCCTCTCGCAAC |
| BC13 | GATCGTTGTGCCAGCACATG |
| BC15 | GATCCTAGTGGGTGCAGATG |
| BC19 | GATCGTTGAGCGTCTCGTTG |
| BC23 | GATCGTACGCTGAGAGGTTG |
