# Supplementary material for: Gene Expression Correlates with the Number of Herpes Viral Genomes Initiating Infection in Single Cells
Source: PLoS Pathog. 2016 Dec 6;12(12):e1006082. doi: 10.1371/journal.ppat.1006082 (PMC5161387; doi:10.1371/journal.ppat.1006082)
Supplement: S4 Table — Sequences for qPCR primers for six viral genes are given, (two from each gene group: immediate-early, early and late genes). A sequence for a cellular housekeeping gene HMBS is given last. (DOCX) [file ppat.1006082.s011.docx]

| **Primer** | **Sequence (5' -> 3')** |
| --- | --- |
| ICP4 fwd | TCAGGTTGTTGCCGTTTATTG |
| iCP4 rev | AAGTTGTGGACTGGGAAGG |
| ICP27 (UL54) fwd | CCTTTCTCCAGTGCTACCTG |
| ICP27 (UL54) rev | GCCAGAATGACAAACACGAAG |
| UL5 fwd | TGTAAAAGGAGATAAGCCCGC |
| UL5 rev | TTGGGTTTAGTGGGACGTTC |
| UL29 fwd | GAAGGTGCATAGGTTACAGGG |
| UL29 rev | GCCAAGATGCTGTTTTACCTG |
| UL19 fwd | GACCGCTTTGTGACTGAGAA |
| UL19 rev | CTGGGTGAGCGTGAAGTTTA |
| US7 fwd | CACGGTCAGTCTGGTATCAA |
| US7 rev | CCCGAGAATAAGCAGGTCTT |
| HMBS fwd | GGCCTGCAGTTTGAAATCAT |
| HMBS rev | CATTCTTCTCCAGGGCATGT |
